# Reducing systematic review burden using Deduklick: a novel, automated, reliable, and explainable deduplication algorithm to foster medical research

**DOI:** 10.1186/s13643-022-02045-9

**Published:** 2022-08-17

**Authors:** Nikolay Borissov, Quentin Haas, Beatrice Minder, Doris Kopp-Heim, Marc von Gernler, Heidrun Janka, Douglas Teodoro, Poorya Amini

**Affiliations:** 1 Risklick AG, Spin-Off, University of Bern, Bern, Switzerland,; 2 CTU Bern, University of Bern, Bern, Switzerland; 3 Public Health & Primary Care Library, University Library of Bern, University of Bern, Bern, Switzerland; 4 Medical Library, University Library of Bern, University of Bern, Bern, Switzerland; 5 University of Applied Sciences and Arts Western Switzerland, Geneva, Switzerland; 6 Department of Radiology and Medical Informatics, University of Geneva, Geneva, Switzerland

**Keywords:** Artificial intelligence, Systematic review, Deduplication, Risklick, Bibliographic databases, Duplicate references, Systematic review software

## Abstract

**Background:**

Identifying and removing reference duplicates when conducting systematic reviews (SRs) remain a major, time-consuming issue for authors who manually check for duplicates using built-in features in citation managers. To address issues related to manual deduplication, we developed an automated, efficient, and rapid artificial intelligence-based algorithm named Deduklick. Deduklick combines natural language processing algorithms with a set of rules created by expert information specialists.

**Methods:**

Deduklick’s deduplication uses a multistep algorithm of data normalization, calculates a similarity score, and identifies unique and duplicate references based on metadata fields, such as title, authors, journal, DOI, year, issue, volume, and page number range. We measured and compared Deduklick’s capacity to accurately detect duplicates with the information specialists’ standard, manual duplicate removal process using EndNote on eight existing heterogeneous datasets. Using a sensitivity analysis, we manually cross-compared the efficiency and noise of both methods.

**Discussion:**

Deduklick achieved average recall of 99.51%, average precision of 100.00%, and average F1 score of 99.75%. In contrast, the manual deduplication process achieved average recall of 88.65%, average precision of 99.95%, and average F1 score of 91.98%. Deduklick achieved equal to higher expert-level performance on duplicate removal. It also preserved high metadata quality and drastically reduced time spent on analysis. Deduklick represents an efficient, transparent, ergonomic, and time-saving solution for identifying and removing duplicates in SRs searches. Deduklick could therefore simplify SRs production and represent important advantages for scientists, including saving time, increasing accuracy, reducing costs, and contributing to quality SRs.

**Supplementary Information:**

The online version contains supplementary material available at 10.1186/s13643-022-02045-9.

## Introduction

Systematic reviews (SRs) and meta-analyses aim to find and synthesize the available evidence within the specific scope of a research question while also striving to minimize bias [1, 2]. Such analyses are time and resource intensive, requiring a median of five researchers and around 40 weeks of work to reach submission [3, 4]. Finding all relevant studies for SRs in health sciences research requires searching multiple bibliographic databases, such as MEDLINE, Embase, Cochrane Library, Web of Science, and Google Scholar and clinical trial registries, such as Cochrane CENTRAL, ClinicalTrials.gov, the International Clinical Trials Registry Platform, and other nation- and region-specific clinical trial registries. Invariably, deduplication is needed to eliminate copies of records [5, 6]. Furthermore, although deduplication of references is essential to ensure the quality of SRs, there is currently no universal method to do so, and the time-intensive task is still mostly performed manually [5].

To economize time spent on deduplication issues, several tools were developed to simplify procedures and increase efficiency [7–11], which require technical proficiency and manual interventions [12]. For example, EndNote [13] is a widely used reference management tool [14] (among others such as Mendeley, RefWorks, and Zotero) offering an integrated deduplication feature, which usually demands careful, multistep configuration procedures [12, 15, 16]. The deduplication process involves long manual procedures, potentially leading to quality-affecting outcome errors [6, 17], such as accidentally removing unique references—a phenomenon that introduces bias [9, 10]. When used alone, EndNote’s sensitivity for deduplication ranges from 51 to 57% [7, 10]. When combined with human validation, adding import filters for various databases, following specific configurations in EndNote, and considering specific order of metadata import increases sensitivity [12].

To address deduplication in SRs, we developed an efficient, reliable, and reproducible deduplication algorithm, Deduklick—a natural language processing (NLP) technology—and compared it with a standardized, manual deduplication process for eight heterogeneous datasets. Deduklick achieved equal to higher expert-level performance on duplicate removal while also preserving high metadata quality and drastically diminishing the time spent on analysis.

## Methods

### Deduplication benchmark datasets

We collected datasets from already executed real-life systematic searches with multiple databases (Table 1). Two different libraries (Public Health & Primary Care Library and Medical Library) performing systematic searches within the University of Bern provided the datasets. The average dataset size was 8078 references. The largest dataset contained 18,314 collected references, while the smallest dataset contained 45 manually collected references. Six datasets (healthy aging, healthy lifestyle, menopause onset, hypertension, e3_gsm, and jugular) contained pooled publications (bibliographic and clinical trial metadata) within the same file.

**Table 1:** Description of datasets used for deduplication analysis

N°	Dataset	Searched databases	References total	Duplicates found by experts	Remaining references
1	Sustainable food	MEDLINE	7595	4438	3157
		Embase Ovid			
		PsycINFO Ovid			
		Web of Science			
		Scopus			
		Lilacs			
		BDENF			
		Google Scholar			
2	Healthy aging	MEDLINE	18,314	7958	10,356
		Embase Ovid			
		PsycINFO Ovid			
		CINAHL			
		Web of Science			
		Cochrane Central			
3	Healthy lifestyle	MEDLINE	13,522	7992	5530
		Embase Ovid			
		Web of Science			
		Cochrane Central			
		Google Scholar			
4	Menopause onset	MEDLINE	8057	4281	3776
		Embase Ovid			
		Web of Science			
		Cochrane Central			
		Google Scholar			
5	Hypertension	MEDLINE	14,024	9478	4546
		Embase Ovid			
		CINAHL			
		Web of Science			
		Cochrane Central			
		ClinicalTrials.gov			
		Epistemonikos			
6	e3_gsm	Medline	1676	1270	406
		Embase Ovid			
		CINAHL			
		Web of Science			
		Cochrane Central			
		ClinicalTrials.gov			
7	Jugular	MEDLINE	1394	1345	49
		Embase Ovid			
		Scopus			
		Cochrane Central			
8	Clinical trials	Cochrane Central, ClinicalTrials.gov, WHO ICTRP	45	15	30

To collect an adequate coverage of scientific evidence, we ran exhaustive searches on multiple databases (Table 1). Each database search contributed a unique set of references; as expected, the combination of searches resulted in high overlap among retrieved references [5]. Type and metadata comprehensiveness differed among databases; thus, they represented other quality metrics to be considered when removing duplicates.

We focused on duplicates from collecting scientific evidence from more than one source of references, including databases for scientific publications as well as clinical trial registries. Following a standardized definition [6, 7, 9], we defined one or more duplicates as an existing unique record having the same title, authors, journal, DOI, year, issue, volume, and page number range metadata.

### Manual deduplication process

Expert information specialists manually deduplicated the datasets based on a defined systematic process using EndNote’s duplicate identification feature. They then changed selection criteria and manually checked for duplicates. They performed the manual deduplication multistep process using EndNote (https://ilias.unibe.ch/goto_ilias3_unibe_cat_2297227.html, “Deduplication in Endnote”). First, they imported all database references into EndNote in a predetermined, specified order. The order of import was based on a well-defined database ranking, which aimed to preserve references with the highest quality metadata (supplementary Table 1). Second, they configured specific field preferences in EndNote. Third, they selected and applied a set of metadata fields, such as title, authors, year, journal, volume, issue, and page number ranges, with EndNote deduplication features. Finally, they ran a semiautomated, rule-based deduplication process with EndNote. To detect and remove duplicates, the EndNote deduplication process involved up to 12 specific combinations of the abovementioned metadata fields. They only executed automatic removal of the duplicates found by EndNote for the first two metadata field combinations as part of the overall 12-step process. To avoid removing unique citations for the remaining 10 steps of field combinations, the information specialists manually eye validated and removed duplicates marked by EndNote.

### Deduklick deduplication process

Since Deduklick (Risklick AG, Bern, Switzerland) automates the deduplication process, we uploaded references for deduplication and subsequently downloaded the results. We formatted (when exported from EndNote) and imported references into Deduklick as RIS files—a format previously developed by the Research Information Systems, Inc. to exchange references between citation management programs. RIS enables a standardized way to exchange references’ metadata among citation programs. Once deduplication is finished, we downloaded an archive that contained deduplication result files and concomitant reports. The result files (RIS format) contained unique references in one folder, and another folder (RIS format) contained removed duplicates. Deduklick provided two reports: (1) flow diagram, which showed pre- and post-deduplication statistics according to PRISMA (Preferred Reporting Items for Systematic Reviews and Meta-Analyses) standards, and (2) the deduplication report, which paired all duplicates with their unique references.

Deduklick’s deduplication used a multistep algorithm of data normalization, calculated a similarity score, and identified unique and duplicate references based on metadata fields, such as title, authors, journal, DOI, year, issue, volume, and page number ranges. Since the syntax of the metadata fields varied among databases, the Deduklick data preprocessor component used NLP technologies to normalize field value into a common representation. Preprocessing (1) removed punctuation and special characters from titles, (2) normalized authors’ and journal names into a common notation, (3) normalized DOI numbers into a common representation by removing URL prefixes and using only DOI identifiers, and (4) harmonized page numbering, among others. As part of preprocessing, non-English titles were translated into English when translation was not part of the metadata. For clinical trials metadata, we extracted other information, such as clinical trial numbers from metadata fields like URL and accession number. As a result of preprocessing, we had a unified and normalized representation of the metadata for publications as well as for clinical trials, ready for deduplication in the subsequent automated step.

Deduklick calculated a similarity score among all references using Levenshtein distance, clustered similar references together, and executed rule-based metadata comparisons of reference fields marking duplicate references. The rule-based decision approach for identifying duplicates is derived from the 12-step manual deduplication multistep process, yet we reduced it to fewer steps to maintain unique references and only remove duplicates. Rather than risk removing unique references when uncertain, we implemented a conservative policy to keep duplicates. To find an efficient threshold for catching duplicates and computational performance, we ran sensitivity analyses with several thresholds within the range ${\theta }_{p}=[60, 95]$, where *p* represents the proximity among all references. We manually cross-compared efficiency and noise between each sensitivity analysis within the range ${\theta }_{p}=[60, 95]$ by measuring numbers of duplicates and time required for deduplication. Ultimately, we selected an optimal threshold of ${\theta }_{p}\ge 78$ based on most favorable efficiency and minimal noise.

### Evaluation method

We compared the algorithm performance with a standardized manual deduplication process using the eight heterogeneous datasets of diverse sizes and research topics. The experts provided us with their datasets, part of executed real-world systematic searches in the past, comprised of metadata from either publications and clinical trials, or a mix of both. For each dataset, duplicates were removed by the experts using the manual deduplication process described earlier. We ran a validation process for each of the eight datasets with goals as follows:

i) Find and eliminate the maximum number of duplicates from already manually deduplicated datasets.
ii) Find erroneously removed unique references and add them back into the validated datasets.

Our validation process resulted in eight benchmark—or gold standard—datasets to compare the efficiency of deduplicating manually vs. deduplicating with Deduklick.

To find additional duplicates and avoid missing false positives (removing unique references unintentionally) and false negatives (remaining duplicates in manually deduplicated datasets), we set low thresholds (${\theta }_{g}\ge 58$) in Deduklick for each of the eight manually deduplicated datasets, where *g* represents the threshold set to find the gold standard (i.e., find bigger clusters of possible lexically similar references). We selected the threshold ${\theta }_{g}\ge 58$ based on sensitivity analyses results within the lower threshold range of ${\theta }_{e}=[50, 80]$. We validated additional duplicates from the eight deduplicated datasets as true duplicates. Subsequently, we found erroneously removed unique references through cross-validation of each of the manually deduplicated datasets with their original raw data before deduplication. We validated and confirmed false positives from each of the eight datasets. We summarize the gold standard cross-validation outcome in Table 2.

**Table 2:** Validated additional duplicates and missing original references in manually deduplicated datasets

Dataset	Validated true duplicates	Validated missing original references
Sustainable food	3	0
Healthy aging	99	6
Healthy lifestyle	104	0
Menopause onset	52	2
Hypertension	364	2
e3_gsm	46	1
Jugular	109	0
Clinical trials	0	0

We measured the performance of each deduplication method using recall, precision, and F1 scores (supplementary Table 2). F1 scores combine recall and precision and represent their harmonic mean. Finally, we measured deduplication task execution time (ET) with Deduklick. Deduplication tasks included preprocessing steps, clustering similar metadata, and removing duplicates based on rules. We excluded time for generating flowcharts and duplicate reports, as well as system roundtrip time for providing results for download.

## Results

Following manual deduplication, we discovered 15–9478 duplicates among the heterogeneous datasets with an average of 4597 duplicates. We present the numbers of additionally validated true duplicates and erroneously removed unique references for the two manually deduplicated datasets in Table 2. In half of the datasets, we found few original references in both groups, which were unintentionally removed after manual deduplication. We validated all additional duplicates as true duplicates; thus, we defined gold standard datasets by adding missing original references and removing additional duplicates from the eight executed datasets (Table 1). With the gold standard datasets, we benchmarked the manual deduplication outcomes with these deduplicated by Deduklick.

Deduklick achieved averages of 99.51% for recall, 100.00% for precision, and 99.75% for F1. In contrast, the manual deduplication process achieved averages of 88.65% for recall, 99.95% for precision, and 91.98% for F1 (Table 3). In six of the eight cases, Deduklick’s F1 score was higher than manual deduplication scores. The clinical trials dataset, which contained 45 selected clinical trials from different sources with corresponding metadata variations, was deduplicated correctly for the manual and the automated processes. These results demonstrate the ability of Deduklick’s algorithmic deduplication to perform at least as good as humans while also avoiding potential errors.

**Table 3:** Comparative table of deduplication results following experts and Deduklick analysis

Dataset	Type	ET s	True +	True −	False +	False −	Recall	Precision	F1
Sustain. food	Experts	4200	3157	4435	0	3	99.91%	100.00%	99.95%
	Deduklick	49	3148	4435	0	12	99.62%	100.00%	99.81%
Healthy aging	Experts	4200	10,356	7853	6	99	99.05%	99.94%	99.50%
	Deduklick	109	10,394	7859	0	61	99.42%	100.00%	99.71%
Healthy lifestyle	Experts	4200	5530	7888	0	104	98.15%	100.00%	99.07%
	Deduklick	92	5592	7888	0	42	99.25%	100.00%	99.63%
Menopause onset	Experts	4200	3776	4227	2	52	98.64%	99.95%	99.29%
	Deduklick	24	3814	4229	0	14	99.64%	100.00%	99.82%
Hypertension	Experts	4200	4546	9112	2	364	92.59%	99.96%	96.13%
	Deduklick	106	4922	9114	0	5	99.90%	100.00%	99.95%
e3_gsm	Experts	4200	406	1223	1	46	89.82%	99.75%	94.53%
	Deduklick	19	447	1224	0	5	98.89%	100.00%	99.44%
Jugular	Experts	4200	49	1236	0	109	31.01%	100.00%	47.34%
	Deduklick	29	159	1236	0	1	99.38%	100.00%	99.69%
Clinical trials	Experts	4200	30	15	0	0	100.00%	100.00%	100.00%
	Deduklick	2	30	15	0	0	100.00%	100.00%	100.00%
*Averages*	Experts	4200	3481.3	4498.6	1.4	97.1	88.65%	99.95%	91.98%
	Deduklick	54	3563.3	4500	0	17.5	99.51%	100.00%	99.75%

On average, the manual deduplication process ET required 70 min or 4200 s; the process was highly dependent on dataset size and information specialist expertise. In comparison, the average Deduklick ET was below a minute for each of the eight datasets. With 18,314 metadata references, the largest dataset (healthy aging) was deduplicated by Deduklick in 109 s, while the smallest dataset (clinical trials) with 45 metadata references was deduplicated in 2 s. We present ET for each dataset in Table 3.

We found 11 false-positive references removed unintentionally from one of the multiple, semiautomated steps with EndNote and manual eye validation; the efficiency of the manual deduplication process depends highly on expertise, experience, and concentration. In the case of Deduklick, we cross-validated the deduplicated references with these from manual deduplication. We observed no false-positive cases among all eight datasets from the Deduklick pool. Deduklick recognized false positives among manual deduplicated datasets as unique references. Therefore, we preserved these false positives in the deduplicated dataset. On average, Deduklick found 82 more duplicates than the information specialists while also demonstrating the highest precision finding unique references. In Table 3, we present true and false positives.

The preferred database rank is another important aspect of deduplication when selecting unique metadata to keep and duplicates to remove. In Table 4, we attribute references to their origin and database. When we found two reference candidates, we reached our decision for removal based on a rule from the defined rank of databases (supplementary Table 1). For all datasets presented in Table 4, we observed an identical distribution of the references before and after deduplication. The differences are mainly due to distinct levels of deduplication performance. In two datasets (healthy aging and healthy lifestyle), we observed a larger shift among the first two ranks, which can be explained by the order of import of the datasets into EndNote (therefore, a human factor). For Deduklick, since the outcome is conserved regardless of reference order in the dataset, the order of reference import is irrelevant.

**Table 4:** Number of deduplicated references ordered by database source

Dataset	Sources	Reference experts	Reference Deduklick	Difference
Sustainable food	MEDLINE	1582	1582	0
	Embase Ovid	291	294	3
	PsycINFO Ovid	334	335	1
	Web of Science	1508	1513	5
	Scopus	477	485	8
	Lilacs	97	94	− 3
	BDENF	1	1	0
	Google Scholar	39	41	2
	Other	109	102	− 7
Healthy aging	MEDLINE	1986	4109	2123
	Embase Ovid	2587	494	− 2093
	PsycINFO Ovid	1164	1207	43
	CINAHL	650	645	− 5
	Web of Science	1388	1284	− 104
	Cochrane Central	183	181	− 2
Healthy lifestyle	MEDLINE	1961	4055	2094
	Embase Ovid	3519	1388	− 2131
	Web of Science	1744	1735	− 9
	Cochrane Central	634	621	− 13
	Google Scholar	100	98	− 2
	Other	34	33	− 1
Menopause onset	MEDLINE	1835	1837	2
	Embase Ovid	1167	1164	− 3
	Web of Science	839	853	14
	Cochrane Central	213	203	− 10
	Google Scholar	99	88	− 11
	Other	128	98	− 30
Hypertension	MEDLINE	3673	3671	− 2
	Embase Ovid	3011	2844	− 167
	CINAHL	195	185	− 10
	Web of Science	1516	1349	− 167
	Cochrane Central	456	447	− 9
	ClinicalTrials.gov	358	360	2
	Epistemonikos	159	152	− 7
	Other	110	94	− 16
e3_gsm	MEDLINE	408	409	1
	Embase Ovid	631	611	− 20
	CINAHL	18	12	− 6
	Web of Science	97	83	− 14
	Cochrane Central	47	44	− 3
	ClinicalTrials.gov	59	60	1
	Other	10	10	0
Jugular	MEDLINE	634	633	− 1
	Embase Ovid	447	367	− 80
	Scopus	155	134	− 21
	Cochrane Central	77	76	− 1
	Other	32	25	− 7
Clinical trials	Cochrane, ClinicalTrials.gov WHO ICTRP	15	15	0

**Fig. 1:**
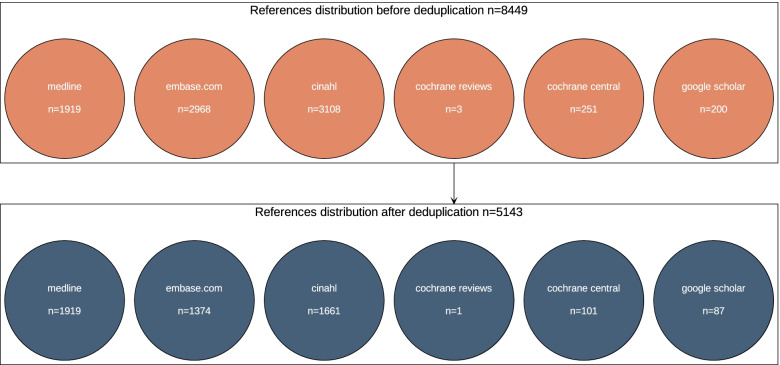
Example of Preferred Reporting Items for Systematic Reviews and Meta-Analyses (PRISMA) deduplication flowchart report following Deduklick analysis

**Fig. 2:**
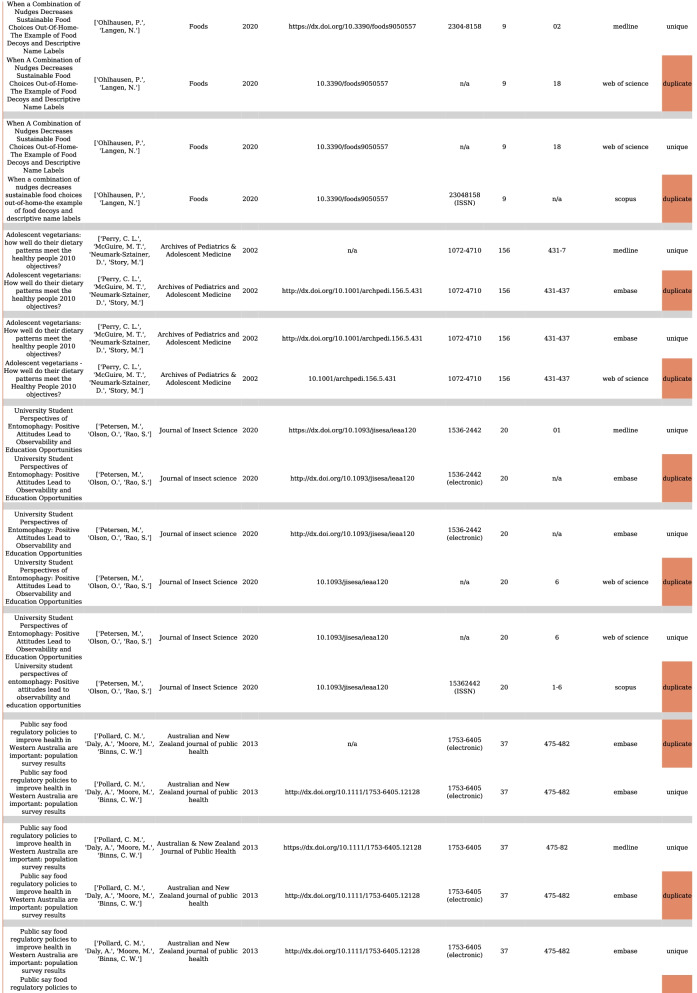
Illustration of deduplication report record with an identified duplicates and corresponding unique reference

Overall, Deduklick performed with an equal to higher quality than manual deduplication while also avoiding false positives and using less time. On average, deduplication with Deduklick required less than a minute on a development machine with 6-CPU-cores and 32 GB of RAM. In Fig. 1, we illustrate the PRISMA flowchart report for the menopause onset dataset before and after deduplication using Deduklick. Figure 2 presents the deduplication report (duplicates versus corresponding unique reference). The PRISMA flowchart report illustrates the distribution of references to database sources before and after deduplication.

## Discussion

After comparing Deduklick’s deduplication algorithm with manual deduplication, we measured performance and required time for eight different datasets. On average, Deduklick’s speed performance was superior to the manual deduplication process. For instance, Deduklick required an average of a minute to perform deduplication, while experts needed an average 70 min to complete the task. In addition to Deduklick’s speed performance, we observed no false positives. Deduklick demonstrated high capabilities for preventing false positives, which we also regularly observed in manual, human-based deduplication processes. In fact, among the datasets, we observed no references removed unintentionally by Deduklick. Deduklick could therefore simplify SRs production and represent important advantages for researchers, including saving time, increasing accuracy, reducing costs, and contributing to quality SRs.

Deduklick is an efficient, reliable, customizable, user-friendly method (supplementary Table 1) based on NLP technologies to detect and remove duplicates. Deduklick was developed to expedite the deduplication process by reducing the technical burden as much as possible with a one-click, software-as-a-service solution. With Deduklick, after uploading files containing metadata from different databases, the results are ready for download in a few minutes. Deduklick is inspired by existing manual deduplication methods requiring expertise and training, such as Bramer’s method [12]; reference management systems, such as Mendeley; and evidence and knowledge synthesis tools from Systematic Review Accelerator, Covidence, and Rayyan. However, these methods still require manual validation of identified duplicates, not to mention important unique references in terms of false positives [3, 9, 10].

Producing SRs requires searching for references in multiple databases and then manually removing duplicates. Logistically, conducting and completing SRs involve significant time and investments in human resources, as well as adequate experience and expertise; on average, SRs require the involvement of five authors or team members and around 40 weeks of work to reach submission [3, 4]. Deduklick’s automatic deduplication method is a robust tool for experts to execute deduplication tasks in a significantly shorter time. In addition to its robust performance (speed and accuracy), Deduklick’s automated method also delineates its decision process when deduplicating and providing transparent reports to validate outcomes. It is reproducible for any kind of dataset; it also provides PRISMA flow diagrams and deduplication reports to meet PRISMA standards. Finally, Deduklick’s dataset deduplication process is scalable; the average deduplication ET is under 1 min. Using the same computer hardware configuration for the datasets, we have run deduplications for larger artificially created datasets containing up to 70,000 references retrieved from multiple databases, where the average execution time of these large datasets was fewer than 10 min.

Adequately reporting applied methods and their results is another important aspect of SRs. According to PRISMA guidelines, authors must cite applied methods for each step of the SR process, including the type and performance of any tools used, as well as a standardized report as a flow diagram of the synthesis process [18, 19]. Since algorithms are often perceived as impenetrable black boxes, comprehensibility, reproducibility of the data transformation processes, and internal decisions and outcomes represent major hurdles when providing AI solutions [20]. However, we describe the Deduklick deduplication process, and its results are evident in downloaded PRISMA flowchart and deduplication reports.

Deduklick’s performance is encouraging. We tested eight EndNote-derived datasets, yet validating additional databases and testing other datasets are required to further explore Deduklick’s full potential. Based on our results, Deduklick’s adaptability for any duplicate search could represent a major impact on professional deduplication approaches. Altogether, Deduklick could become a preeminent performant and reliable deduplication solution. Data professionals who use and adopt Deduklick for such tasks redeem time, enhance performance, drastically diminish production costs, and increase the quality of all deduplication-associated procedures. Deducklick also expedites medical research by automating the time- and resource-intensive deduplication process for SRs.

## Supplementary Information

**Additional file 1. **Ranking table of databases used in deduplication analysis.

**Additional file 2. **Definition and calculation methods applied to evaluate deduplication procedures.

## Data Availability

Data from the deduplication cohorts will be made available upon reasonable request. For access, please email the corresponding author. The code used in the development of our model will not be shared because we present in detail the methods used in the model development.

## Abbreviations

AI: Artificial intelligence
ET: Execution time
NLP: Natural language processing
PRISMA: Preferred Reporting Items for Systematic Reviews and Meta-Analyses
RIS: Research information systems
SR: Systematic reviews
