# Supplementary material for: Reducing systematic review burden using Deduklick: a novel, automated, reliable, and explainable deduplication algorithm to foster medical research
Source: Syst Rev. 2022 Aug 17;11:172. doi: 10.1186/s13643-022-02045-9 (PMC9382798; doi:10.1186/s13643-022-02045-9)
Supplement: Supplementary file 1 — Additional file 1. Ranking table of databases used in deduplication analysis. [file 13643_2022_2045_MOESM1_ESM.pdf]

Database Ranking	
**Rank**	**Database name**
1	Medline
2	Embase
3	Psycinfo
4	Eric
5	Pubmed
6	Cinahl
7	Web of Science
8	Scopus
9	Proquest
10	Cochrane Reviews
11	Cochrane Central
12	Lilacs
13	Global Index Medicus
14	African Index Medicus
15	SciELO
16	ClinicalTrials.gov
17	WHO ICTRP
18	Dimensions
19	Epistemonikos
20	Google Scholar
