# Supplementary material for: Reducing systematic review burden using Deduklick: a novel, automated, reliable, and explainable deduplication algorithm to foster medical research
Source: Syst Rev. 2022 Aug 17;11:172. doi: 10.1186/s13643-022-02045-9 (PMC9382798; doi:10.1186/s13643-022-02045-9)
Supplement: Supplementary file 2 — Additional file 2. Definition and calculation methods applied to evaluate deduplication procedures. [file 13643_2022_2045_MOESM2_ESM.pdf]

Metric	Description
True Positives	Number of correctly identified duplicate references removed from the dataset
True Negatives	Number of references that should and do remain present in the dataset
False Positives	Unique references wrongly identified as duplicates and removed from the dataset
False Negatives	References which remain in the dataset, but should have been removed as duplicates
Precision	$\frac{True\ Positives}{True\ Positives\ +\ False\ Positives}$
Recall (Sensitivity)	$\frac{True\ Positives}{True\ Positives\ +\ False\ Negatives}$
F_1 Score	$\frac{2 * Precision * Recall}{Precision\ +\ Recall}$
